# Inflammation as an orchestrator of cutaneous scar formation: a review of the literature

**DOI:** 10.20517/2347-9264.2020.150

**Published:** 2020-10-16

**Authors:** Traci A. Wilgus

**Affiliations:** Department of Pathology, Ohio State University, Columbus, OH 43210, USA.

**Keywords:** Inflammation, scar, skin, wound healing, macrophage, mast cell, neutrophil

## Abstract

Inflammation is a key phase in the cutaneous wound repair process. The activation of inflammatory cells is critical for preventing infection in contaminated wounds and results in the release of an array of mediators, some of which stimulate the activity of keratinocytes, endothelial cells, and fibroblasts to aid in the repair process. However, there is an abundance of data suggesting that the strength of the inflammatory response early in the healing process correlates directly with the amount of scar tissue that will eventually form. This review will summarize the literature related to inflammation and cutaneous scar formation, highlight recent discoveries, and discuss potential treatment modalities that target inflammation to minimize scarring.

## INTRODUCTION

The repair of wounds in mature skin has been studied extensively. This process is highly complex and interactive, and it is made up of a series of well-defined stages that include inflammation, proliferation, and remodeling/scar formation^[1–3]^. Scar tissue is generated from activated fibroblasts, which produce excess levels of irregularly organized collagen. Clinically significant scars can develop from surgical, traumatic, or thermal (e.g., burn) injury^[4]^. Scars essentially function as a quick patch for damaged dermal tissue, but they can be problematic in many ways. Compared to normal skin, scar tissue is weaker in terms of tensile strength and it is also more rigid^[5,6]^, which adversely affects the biomechanical properties of the skin. The replacement of normal tissue with scar tissue reduces the functional capacity of the skin in that area as well, and this can cause problems such as limiting joint motion and impairing normal tissue growth. Scars can also have significant psychosocial implications and can negatively impact a patient’s quality of life^[7]^.

Although inflammation occurs relatively early after injury, it can impact later stages of repair such as scar formation. In the early stages of repair, the primary functions of inflammation are to clean the wound site, clear debris, and prevent infection^[8]^. However, many of the mediators produced by activated inflammatory cells can stimulate fibroblasts, which drives the production of scar tissue and consequently shapes the final outcome of repair. Several innate immune cells have been linked to scar formation, including neutrophils, mast cells, and macrophages. In addition, a variety of inflammatory mediators have been shown to influence scar formation. This review will summarize what is known about the role of inflammation in scar formation and discuss some potentially useful approaches to reduce scar formation by modulating inflammation.

## CORRELATIVE DATA LINKING INFLAMMATION AND SCARRING

**Figure 1.**
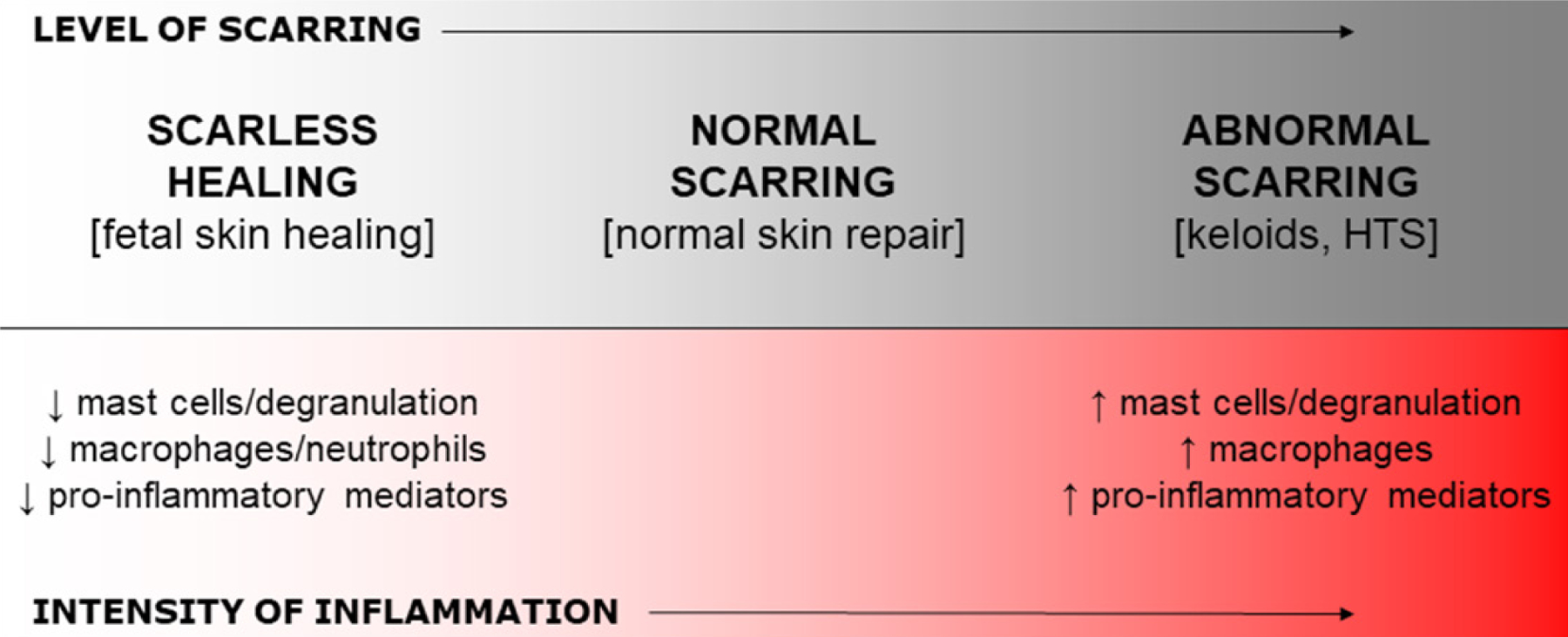
Summary of the relationship between scarring and inflammation. Most studies published to date indicate that the robustness of the inflammatory response resulting from skin injury correlates with the amount of scar tissue that will be produced, with low levels of inflammation in scarless wounds and high levels of inflammation in cases of abnormal or excessive scarring

A large number of studies suggest that the level of inflammation in injured skin correlates directly with scar formation. A direct association between the extent of injury (and hence the levels of inflammation) and the amount of scarring that will ultimately result from the wound healing process has been established. For example, larger/deeper wounds or injuries that cause more severe damage are associated with higher levels of inflammation and heal with more scar tissue^[9–13]^. Furthermore, a wide range of studies have shown that high levels of inflammation are associated with excessive scarring or abnormal scars such as keloids and hypertrophic scars (HTS), whereas inflammation is significantly blunted in wounds that heal without scars [Figure 1]. Evidence supporting this concept will be discussed throughout the remainder of this section.

### Abnormal scars

#### Keloids

Keloids are abnormal, raised scars that develop after injury. These scars display some similarities with tumors, as they tend to invade the adjacent skin and extend beyond the initial site of injury. There are no widely accepted experimental animal models of keloid disease, so studies on keloid pathogenesis are generally limited to experiments on tissue from human patients. A number of studies have examined inflammation in keloid tissue and the vast majority have indicated that keloids are associated with an increase in pro-inflammatory mediators and inflammatory cells.

Several studies have suggested an increase in pro-inflammatory mediator expression in keloids. A study by Zhang *et al*.^[14]^ suggested that a pro-inflammatory niche exists in keloids, based on observations that interleukin (IL)-6 and IL-17 were increased in keloid tissue compared to normal skin. Another group reported an increase in chemokine-like factor 1 and other pro-inflammatory cytokines, such as IL-6, IL-8, and IL-18, in keloid tissue compared to normal skin and normal scars^[15]^. Jumper *et al.*^[16]^ performed a site-specific gene expression profiling study and found that keloid tissue was enriched for pro-inflammatory genes and pro-inflammatory signaling pathway members, including IL-1β, IL-8, and IL-17, among others. Interestingly, the data pointed to a possible role for the overlying epidermal cells in keloid lesions in the regulation of inflammation.

In addition to pro-inflammatory mediators, several inflammatory cell types are prominent in keloids, particularly mast cells and macrophages. A study by Dong *et al.*^[17]^ suggested that the total number of mast cells and the number of mast cells expressing chymase, a serine protease found primarily in mast cells, are higher in keloid tissue compared to normal skin. An increase in chymase activity in keloids was also observed, and chymase was shown to stimulate collagen production in cultured fibroblasts^[17]^. Another study found a low number of mast cells expressing tryptase, another mast cell-related serine protease, in keloids compared to tissue from several other organs; however, the sample size was small (*n* = 3 keloids) and there was no direct analysis of or comparison to normal skin^[18]^. Several studies have also shown an increase in macrophages in keloids compared to normal skin^[19,20]^. A study by Shaker *et al.*^[21]^ showed that both macrophages and mast cells are frequently found in close proximity to fibroblasts in keloid tissue, and another study by Arbi *et al.*^[22]^ showed a close association between mast cells and fibroblasts in keloid samples by transmission electron microscopy. Although these studies were limited in scope, they do suggest the possibility that direct cell-cell interactions between inflammatory cells and fibroblasts could be important for fibrosis.

A more comprehensive analysis of inflammatory and immune cells was performed by Bagabir *et al.*^[23]^ In this study, the number of mast cells and degranulated (activated) mast cells were found to be increased in keloids compared to normal skin and normal scar tissue, as were the number of M1 and M2 macrophages. M1 (classically activated; pro-inflammatory) and M2 (alternatively activated; anti-inflammatory/pro-fibrotic) macrophages express different biomarkers, and this general classification system is commonly used to delineate different macrophage phenotypes^[24]^. Interestingly, inflammatory cell enrichment was more closely associated with intralesional and perilesional keloid sites as opposed to extralesional sites.

While most published studies indicate an increase in mast cells and macrophages in keloid scars, one study comparing human keloids and equine exuberant granulation tissue (a fibrotic condition suggested to have some similarities to human keloids) reported minimal mast cells and macrophages in both fibrotic conditions^[25]^. Two other studies suggested either minimal differences or fewer mast cells in keloids^[26,27]^. While the reasons for the discrepancies between the studies are not entirely clear, the observations by Bagabir *et al.*^[23]^ described earlier showed that the number of inflammatory cells varied across different sites within a keloid lesion. Also, in some of the studies, granule markers were the sole staining method used, which could underestimate the number of mast cells if they have degranulated. Therefore, the specific locations at which the cells were quantified, in addition to differences in staining methods, sample sizes, and patient populations, could contribute to the inconsistent results.

#### HTS

HTS, which are raised red scars, also fall into the category of abnormal scars. Unlike keloids, HTS are confined to the borders of the original wound. A wide range of studies in both small and large animal models as well as human clinical samples have examined inflammation in hypertrophic scarring.

Several rodent models of HTS have been described. One such model uses the application of a mechanical loading device to apply tension to wounds, inducing a HTS-like phenotype reminiscent of human HTS^[28]^. In this model, the scars were shown to have a high number of mast cells, similar to human HTS^[28]^. The HTS-like lesions were also shown to have higher numbers of macrophages compared to control wounds with no mechanical loading^[29]^. Several other studies have been published using models of HTS formation in which human skin is grafted onto nude mice. In these studies, higher numbers of mast cells^[30]^ and macrophages^[30,31]^ were observed in the HTS-like lesions compared to control tissue. Upon further examination of macrophage subtypes, M2 macrophages were found to be elevated, and higher levels of pro-inflammatory cytokines were noted in HTS-like samples^[31]^. An increase in both mast cells and macrophages has also been reported in a mouse model of hypertrophic scar contracture^[32]^.

Large animal models, such as pigs, are considered by many to be an ideal wound healing model based on similarities in skin anatomy between pig and human. A study by Harunari *et al.*^[33]^ examined mast cells in human HTS samples and samples from a large animal model of HTS, the red Duroc pig. The authors found an increase in mast cells in HTS from both humans and red Duroc pigs compared to the corresponding normal skin controls.

In human HTS, there are conflicting results regarding whether inflammation is enhanced or reduced in HTS. Early studies by Kischer *et al.*^[34]^ reported higher numbers of mast cells in HTS compared to granulation tissue or mature scar tissue samples using toluidine blue, a metachromatic stain that binds mast cell granules. A study by Beer *et al.*^[35]^ did not see differences in tryptase-positive mast cells when comparing among keloids, HTS, and surgical scars; however, normal skin samples were not included for comparison. Another study by Niessen *et al.*^[36]^ found no significant differences in tryptase-positive mast cells in HTS compared to normal scars, although they did note a trend toward increased subepidermal mast cells in HTS. There are several possible explanations for the contradictory results between the studies. The use of different techniques to identify mast cells could have played a role, as toluidine blue should indiscriminately stain all mast cell subtypes (including chymase- and tryptase-positive mast cells), whereas tryptase staining may not easily identify mast cells if they predominately express chymase. In addition, the age of the scars examined may play a role. The study by Beer *et al.*^[35]^ showed a direct correlation between mast cell density and scar age in surgical scar samples and the study by Niessen *et al.*^[36]^ noted an overall increase in mast cells between 3 and 12 month scar samples, so standardization of scar age may be needed to properly compare studies from different investigators.

There are only a few reports examining macrophages in human HTS. In one study comparing normal scars and HTS from breast surgeries, no differences were found in macrophages between scar types^[36]^. In another study looking at scars from cardiothoracic surgery patients, an increase in macrophages was observed in normal scars compared to HTS at earlier time points, but total macrophage and M2 macrophage numbers were increased in HTS at later time points and remained elevated for a longer period of time^[37]^.

The data on pro-inflammatory cytokine expression in human HTS also appears to be somewhat mixed. One study compared inflammatory gene expression in a small prospective study comparing patients that developed normal scars or HTS^[37]^. The authors reported reduced expression of inflammatory genes (including TNFα, IL-1α, IL-1RN, several chemokines, and IL-10) in HTS, but the downregulated genes contained both pro- and anti-inflammatory genes. Similarly, another prospective study compared various parameters of inflammation at a very early time point (3 h post-injury) in patients that healed with normal scarring or HTS^[38]^. The authors reported reduced protein levels of IL-6, IL-8, and CCL2 in wounds from HTS patients compared to normal scar patients and suggested that reduced inflammation is associated with HTS formation. However, increases in P-selectin mRNA were found in HTS compared to normal scar samples. In addition, TNF-α, CXCL4, VCAM, and TLR4 mRNA were significantly increased after surgery only in HTS and there were more M2 macrophages in pre-operative HTS samples.

Taken together, the data suggest that more detailed analysis of inflammatory cells and inflammatory mediators is needed to definitively show whether inflammation is associated with hypertrophic scarring in humans.

### Scarless healing

#### Fetal skin wounds

It is well established that developing fetal skin is capable of healing wounds in a scarless, regenerative manner at certain gestational stages. Fetal skin heals scarlessly at early stages of development (until about the third trimester), but as the skin matures at later stages of development it heals through a fibrotic repair process that produces a permanent scar^[39–41]^. One of the key differences between scarless and fibrotic fetal wound healing is the level of inflammation. Many studies have shown that there are few, if any, of the traditional features of inflammation in scarless fetal wounds, but fibrotic fetal wounds that occur later in gestation heal with a strong inflammatory response^[42]^. In addition, artificially inducing inflammation in fetal wounds that would normally heal scarlessly causes them to heal with a scar^[43]^.

Several studies have been performed to identify specific differences in the inflammatory response between scarless and fibrotic wounds. Levels of pro-inflammatory mediators, such as lipids (PGE_2_)^[44]^, cytokines (IL-6, IL-8, IL-33)^[45–47]^, and alarmins (HMGB-1)^[48]^ have been shown to be lower while anti-inflammatory mediators (IL-10)^[49]^ has been shown to be higher in fetal skin or scarless wounds. Fetal fibroblasts produce less IL-6 and IL-8 compared to adult fibroblasts^[45,46]^ and microarray studies showed that expression of inflammatory genes is reduced in fibroblasts from less developed fetal skin (scarless) compared to more developed fetal skin (fibrotic)^[50]^.

In addition to reduced pro-inflammatory mediators, fewer inflammatory cells or reduced inflammatory cell activation have been described in scarless fetal wounds. Fetal platelets do not aggregate to the same extent and release lower levels of cytokines compared to adult platelets^[51,52]^. Mast cells are present in lower numbers and are less mature in fetal skin at earlier gestational ages, and mast cells do not become activated or degranulate in response to injury in scarless wounds^[53,54]^. Macrophages are fewer in number and persist for a shorter period of time in early embryonic wounds, and the macrophages that are present do not appear to be activated^[55,56]^. In addition, very few, if any, neutrophils are recruited to fetal wounds when the skin is injured at ages corresponding to scarless healing^[42,57]^. It is possible that differences in fetal endothelial cells could play a role in minimizing the number of circulatory inflammatory cells recruited to fetal wounds. Studies have shown that neutrophils adhere less to fetal endothelial cells, which is likely due to lower expression of P-selectin^[58,59]^. This adhesion molecule mediates leukocyte-endothelial cell interactions that are required for effective recruitment of leukocytes from the circulation and into damaged tissues.

Studies examining human fetal skin also support the idea that less developed skin has a suppressed inflammatory response. An early study by Rowlatt described a lack of an acute inflammatory reaction and granulation tissue formation at the site of limb amputations caused by amniotic constriction bands in a 20 week human fetus^[60]^. In another study, Walraven *et al.*^[61]^ reported that mid-gestation fetal skin (18–22 weeks) has fewer macrophages, mast cells, dendritic cells, and other immune cell types compared to adult skin. Because there was an adequate number of immune cells in the lymph nodes, the authors speculated that the reduced immune cell numbers present in the skin was due to a deficiency in homing signals. Indeed, they found reduced levels of chemokines such as CCL17, CCL21, and CCL27 in fetal skin.

#### Other types of scarless wounds

Besides fetal skin, scarless healing has also been described in mucosal tissues, such as the oral mucosa^[62]^. There is also evidence that oral mucosal wounds have a blunted inflammatory response compared to other tissues that heal with scarring. In a mouse model, Szpaderska *et al.*^[63]^ showed fewer macrophages, less myeloperoxidase (a marker of neutrophil presence), and lower pro-inflammatory cytokine production in oral mucosal wounds (scarless) compared to cutaneous wounds (scar-forming). Similarly, in a porcine model, Mak *et al.*^[64]^ compared oral and skin wounds in red Duroc pigs and found reduced macrophage and mast cell numbers in oral wounds, which also displayed accelerated resolution of inflammation. In healthy human oral and skin tissue (uninjured), fewer neutrophils and macrophages have been reported^[65]^. Global transcriptome analysis by multiple groups suggests that several pro-inflammatory genes and regulatory pathways are suppressed or are less persistent in oral mucosal wounds compared to skin wounds^[66,67]^.

Scarless healing has also been documented in a unique mouse species, the African spiny mouse (*Acomys*). *Acomys* was shown to completely regenerate and heal without scarring in response to large dorsal skin wounds^[68]^. Follow up studies compared transcription profiles of scar-forming wounds from standard laboratory mice (*Mus*) and regenerative wounds from *Acomys*, and showed *Acomys* wounds had a diminished cytokine/chemokine response^[69]^. *Acomys* has also been reported to be neutropenic (reduced blood neutrophils) and have less pronounced macrophage recruitment to wounds compared to *Mus* strains^[70]^. These results fit the general theme observed with scarless oral mucosal and fetal wounds, which have a dampened inflammatory response compared to wounds that heal with scars.

## FUNCTIONAL DATA LINKING INFLAMMATION AND SCARRING

In addition to correlative data, which generally link higher inflammatory mediator levels and/or elevated inflammatory cell numbers in a wound to more abundant scar formation, there are also functional data supporting a role for inflammation in promoting scar formation. These include studies showing that inflammatory cells or inflammatory mediators stimulate scar tissue/collagen production as well as studies showing that depletion or knockdown of inflammatory components reduces scar formation.

### Inflammatory cells

#### Mast cells

Two general approaches have been used to study the function of mast cells in wound healing and scar formation: treatment with mast cell stabilizing drugs and examination of mast cell-deficient mouse strains.

Drugs that act as mast cell stabilizers, which prevent mast cell degranulation, have been used in animal models of wound healing to study the importance of mast cells in scar formation. By preventing mast cell degranulation, these drugs inhibit the release of pre-stored mediators present within the granules. Disodium cromoglycate (also known as cromolyn) has been shown to reduce collagen content in a rat model when injected directly into wounds^[71]^. Systemic treatment with disodium cromoglycate has also been shown in a mouse excisional wound model to reduce pro-inflammatory cytokine levels and myeloperoxidase levels (commonly used to estimate the presence of neutrophils) at early time points post-injury, while reducing scar size and normalizing collagen architecture/collagen fibril density at later time points^[72]^. Another study showed that oral administration of the mast cell stabilizer ketotifen reduced wound contraction and collagen deposition, causing thinner, less dense collagen fibrils to be produced in a red Duroc pig model^[73]^.

The role of mast cells in scar formation has also been examined in mast cell-deficient mouse strains (either naturally occurring mutants or genetically modified mice), and the results seem to differ depending on the mouse strain and wound model used. Kit^W/W-v^ mice are mutant mice that lack mast cells due to functional mutations in the tyrosine kinase receptor c-kit, which binds an important growth factor for mast cells (stem cell factor). Younan *et al.*^[74]^ used these mice in a study looking at the effects of microdeformation in wounds using a negative pressure wound therapy device. They showed that microdeformation induced higher levels of mast cell degranulation, which correlated with an increase in granulation tissue thickness and collagen deposition. These changes were normalized in Kit^W/W-v^ mice, suggesting that the increases in granulation tissue and collagen production from the microdeformation device were mast cell-dependent. Kit^W/W-v^ mice were also shown to heal with smaller scars in late-gestation fetal skin wounds compared to control mice^[53]^ and less fibrosis at the wound edge in adult scald wounds^[75]^, supporting the idea that mast cells promote scar formation and fibrosis. In contrast to these studies, several other mast cell-deficient mouse strains have been shown to heal with similar levels of scar tissue and collagen deposition compared to control mice that have normal mast cell numbers^[76–78]^. Many of the published studies discussed above used different wound models and different mast cell-deficient strains, which could partially explain the variable results. In addition, most mast cell-deficient strains also have defects in one or more other immune cell types and non-specifically deplete the entire mast cell population, so it will be important to revisit these ideas once we understand more about mast cell heterogeneity and have more precise mouse models to specifically target mast cells and possibly different functional mast cell subtypes^[79]^.

#### Neutrophils and macrophages

Several mutant mouse strains have been used to examine the importance of neutrophils and macrophages in wound-induced scar formation. One of the first studies of this kind explored wound healing in mice lacking the transcription factor PU.1^[80]^. PU.1 null mice, which lack macrophages and functional neutrophils, were shown to heal quickly and with minimal scarring. However, the relative importance of macrophages versus neutrophils is unclear since both cell types are absent in wounds from PU.1 null mice. While some studies suggest that the early neutrophil response may be important for scar formation and the presence of neutrophil extracellular traps have been reported in skin scars and other fibrotic conditions^[81]^, there do not appear to be published studies examining scar formation using animal models in which neutrophils have been specifically depleted.

More specific studies examining the role of macrophages in collagen deposition and scar formation have been performed using several approaches. Studies from two groups have used slightly different genetically modified mouse strains in combination with diphtheria toxin to deplete macrophages. Mirza *et al.*^[82]^ showed reduced collagen density in wounds from macrophage-depleted mice and Lucas *et al.*^[83]^ showed that depletion of macrophages at early stages of wound healing reduced the amount of granulation tissue and the size of scars that formed. Several studies have also used clodronate liposomes to deplete macrophages. One study showed that macrophage depletion reduced collagen expression and deposition in wounds^[84]^ and another showed that macrophage depletion reduced scar formation in a xenograft model of HTS^[85]^. While it is generally accepted that macrophages release mediators that can stimulate collagen production by fibroblasts, several recent studies have suggested the possibility of novel mechanisms. For example, macrophages were shown to support adipocyte-derived myofibroblasts through insulin-like growth factor and platelet-derived growth factor C^[86]^. A recent report also highlighted the plasticity of myeloid cells, which may be converted directly to collagen-producing fibroblasts in healing wounds^[87]^. More work will have to be done to understand exactly how macrophages contribute to scar formation.

### Inflammatory mediators

Aside from specific inflammatory cell types, a wide range of inflammatory mediators, including both pro- and anti-inflammatory mediators, have been shown to play a functional role in cutaneous scar formation.

#### Pro-inflammatory mediators

Both fetal and adult wound healing studies in animal models have implicated specific pro-inflammatory mediators in the stimulation of scar formation. Fetal wound healing models have been useful for identifying inflammatory mediators with fibrogenic potential since a specific mediator can be injected into fetal wounds to determine whether it can convert the scarless healing process into a fibrotic repair process. With this approach, the formation of a scar in a wound that would otherwise heal scarlessly can be used as a readout of pro-fibrotic activity. A number of pro-inflammatory mediators have been shown to promote scar formation in fetal wounds using this system, including cytokines such as IL-6 and IL-33^[45,47]^, pro-inflammatory lipids like PGE_2_^[44]^, and alarmins such as HMGB-1^[48]^. Some of these same mediators have also been studied in adult wound healing models. For example, HMGB-1 has been linked to scar formation^[88]^ and blocking PGE_2_ production with drugs that inhibit cyclooxygenase-2 activity has been shown to reduce scar formation in adult incisional wound models^[89,90]^. Other pro-inflammatory cytokines that have been linked to cutaneous scar formation and/or collagen production in adult scar/fibrosis models include IL-17^[91]^ and monocyte chemoattractant protein-1^[92,93]^. Additionally, osteopontin (OPN), which has pro-inflammatory cytokine-like properties and is associated with wound-induced inflammation^[94]^, has been linked to scar formation. Studies in a mouse model showed that osteopontin knockdown in the skin resulted in less inflammation (reduced neutrophil, macrophage, and mast cell numbers) as well as reduced scar formation compared to control wounds^[95]^.

#### Anti-inflammatory mediators

In contrast to pro-inflammatory mediators, anti-inflammatory mediators have been shown to limit scar formation in fetal and adult wound healing models. The most well-documented example of this is the anti-inflammatory cytokine IL-10. Studies have shown that IL-10 levels are higher in scarless fetal wounds compared to scar-forming wounds^[49]^. Furthermore, scar formation is amplified in IL-10 knockout mice^[96]^ and scar formation is reduced when IL-10 levels are artificially enhanced^[11,49,97–99]^. In addition to IL-10 having anti-inflammatory effects, studies have suggested that IL-10 signaling enhances the production of hyaluronic acid^[100–103]^, an extracellular matrix molecule associated with scarless healing and regeneration^[104–110]^. Other anti-inflammatory and pro-resolution mediators have also been linked to scar formation. Mice lacking the chemokine receptor CXCR3, which has been implicated in wound resolution, heal with abnormal scarring^[111]^, and the pro-resolution mediator chemerin15 has been shown to reduce inflammation and scar formation^[112]^.

## THERAPEUTIC STRATEGIES TO PREVENT SCAR FORMATION

Given the evidence supporting the idea that inflammation promotes scar formation, it seems logical that targeting inflammation might be a viable therapeutic strategy for restricting scar tissue production and enhancing the cosmetic and functional clinical outcomes resulting from skin injury. There are data supporting various anti-inflammatory approaches that could be effective for minimizing the appearance of scars and several other proposed scar therapies may reduce scarring in part by altering inflammation. These will be discussed below.

### Traditional anti-inflammatory strategies

Steroids and nonsteroidal anti-inflammatory drugs (NSAIDs) are traditional anti-inflammatory drugs that may be beneficial for preventing or treating scars. Corticosteroids are anti-inflammatory drugs commonly used in clinical settings to treat raised scars such as HTS and keloids. Steroid therapy can be used in an attempt to induce scar regression, but may be more effective when used to prevent recurrence after scar revision surgery^[113]^. The use of steroids for scar therapy has been reviewed previously^[114–116]^.

NSAIDs are another class of anti-inflammatory drugs that block the production of inflammatory lipid mediators, such as PGE_2_, by inhibiting the function of one or more cyclooxygenase enzymes. These drugs are commonly used to treat pain, fever, and inflammation. Some of these drugs have been suggested to reduce collagen deposition and scar formation in animal models of wound healing. In an incisional murine wound model, topical application of celecoxib resulted in decreased PGE_2_ production, inhibition of neutrophil recruitment, and significantly reduced scar size^[89]^. In a rabbit ear model of HTS formation, celecoxib treatment was shown to reduce scarring (measured by scar elevation index score)^[90]^, and a combination of celecoxib and the angiotensin-converting enzyme inhibitor captopril reduced inflammation and scar height^[117]^. Studies have also suggested that dressings incorporated with anti-inflammatory drugs, such as electrospun fibrous scaffolds loaded with ibuprofen^[118]^ and emulgel dressings containing the anti-inflammatory drug acetylsalicylic acid along with stratifin^[119]^ (a protein produced by keratinocytes that has been shown to suppress scar formation^[120,121]^), may lead to reduced scarring.

### Other anti-inflammatory strategies

#### TLR4 inhibitors

Drugs that target innate immune receptors have the potential to be used to limit scar formation based on their anti-inflammatory mechanism of action. TLR4 inhibitors are one example. TLR4 is a pattern recognition receptor that binds to many different pathogen-associated molecular patterns as well as damage-associated molecular patterns (also known as alarmins) that stimulate inflammation in response to microbes or tissue damage, respectively^[122]^. High TLR4 expression has been documented in human HTS tissue and HTS fibroblasts^[123]^. TLR4 has also been suggested to play a role in HTS development in a mouse model, where treatment with the TLR4 inhibitor TAK-242 (restorvid) was found to reduce scar formation^[124]^. The data were very limited in this study, with only 3 mice per group and no quantitative scar data; however, TLR4 has been linked to fibrosis previously and TAK-242 has been shown to reduce fibrosis in several organs, including the skin, in other animal studies^[125–127]^. Together, these studies suggest TLR4 inhibitors may be a promising strategy to minimize scarring. In addition, the fact that this drug has been used topically to reduce ultraviolet light-induced inflammation and skin carcinogenesis^[128,129]^ and has been used in clinical trials for other diseases^[130]^, suggests that TLR4 inhibitors may be a worthwhile pursuit.

#### CXCR4 antagonists

Another potential target is the chemokine receptor CXCR4, which binds to stromal-derived factor-1 (SDF-1), also known as CXCL12. Upregulation of SDF-1/CXCR4 signaling has been reported in human burn patients and in HTS tissue^[131,132]^. An increase in SDF-1 is believed to stimulate recruitment of CXCR4-positive leukocytes, and possibly collagen-producing fibrocytes, from the circulation, thereby promoting HTS formation^[131,132]^. Similar results have been reported in keloid tissue, with an increase in SDF-1 expression and CXCR4-positive cells in keloids compared to normal tissue^[133]^. In a small animal HTS model, a CXCR4 antagonist was found to reduce the number of macrophages and myofibroblasts, inhibit contraction, and reduce scar formation^[131]^. Although more work is needed, the results thus far suggest potential for CXCR4 antagonists in preventing scar tissue deposition.

### Additional scar reducing treatments affecting inflammation

#### Pirfenidone

Pirfenidone is an anti-fibrotic drug with anti-inflammatory properties used to treat idiopathic pulmonary fibrosis. Although the ability of pirfenidone to treat or prevent skin scarring has not been thoroughly investigated, several studies suggest that it may be a promising option. Multiple studies have shown that pirfenidone can inhibit the pro-fibrotic behavior of cultured dermal fibroblasts. For example, pirfenidone can reduce TGF-β-induced fibroblast proliferation, migration, collagen expression, and myofibroblast formation^[134]^, as well as fibroblast contraction^[135,136]^. Pirfenidone has also been shown to reduce proliferation and inhibit epithelial-mesenchymal transition in keloid keratinocytes^[137]^. In wound healing studies, pirfenidone has been shown to reduce pro-inflammatory cytokine production, neutrophil infiltration, and collagen synthesis^[138,139]^, and in a clinical trial topical application of an 8% pirfenidone gel induced scar regression to a greater degree than control pressure therapy in burn-induced HTS in pediatric patients^[140]^.

#### Epigallocatechin-3-gallate

Epigallocatechin-3-gallate (EGCG) is a green tea polyphenol known to have antioxidant, anti-inflammatory, and anti-tumor effects. The ability of EGCG to be photoprotective and inhibit cutaneous inflammation in response to ultraviolet light-induced skin damage is well documented^[141–144]^. More recently, the potential for EGCG to inhibit inflammation and scar formation in skin wounds has been described. EGCG has been shown to inhibit mast cell-stimulated collagen protein expression by keloid fibroblasts, likely through alterations in PI3K/Akt signaling^[145]^. EGCG also inhibited proliferation, migration, and collagen expression in keloid fibroblasts via STAT3 inhibition^[146]^. In a study using a model in which human keloid fibroblasts were injected into nude mice, EGCG treatment reduced keloid nodule formation and inhibited collagen production^[146]^. Another study tested the effects of EGCG in long-term keloid organ cultures and showed that EGCG reduced mast cell numbers in the keloid tissue and reduced keloid volume^[147]^. Recently, a clinical trial examining the effects of a topical formulation of EGCG in human skin wounds showed that EGCG treatment reduced mast cell numbers and improved scar outcomes, as indicated by a reduction in scar thickness and increases in hydration and elasticity^[148]^. Together, the data suggest that EGCG could be a promising therapeutic to prevent excessive scarring.

#### Fibromodulin

Fibromodulin is a member of the small leucine-rich proteoglycan family that has been shown to have an inverse relationship with scar formation. Fibromodulin levels are initially high in fetal skin at stages associated with scarless healing, then levels decrease during development as the skin starts to heal with a scar^[149]^. Additionally, fibromodulin levels increase during healing in scarless fetal wounds, which have minimal inflammation, but remain low in scar-forming wounds which display a typical inflammatory response^[149]^. Lower fibromodulin levels have been reported in HTS from human subjects and animal models compared to normal tissue^[150,151]^, and mice lacking fibromodulin heal with more inflammation^[152]^ and more scar formation^[153,154]^. Increasing fibromodulin levels via adenoviral overexpression or treating with recombinant fibromodulin has been shown to reduce scar formation in rabbit, mouse, and red Duroc pig wounds^[153–156]^. Based on these studies, a fibromodulin-based peptide has been developed and is currently being tested in clinical trials^[157]^.

#### Hydration

Studies have shown that maintaining hydration and allowing healing to occur in a moist environment can be beneficial for healing and reducing scar formation. The underlying mechanisms appear to be related to reduced inflammation. Saline-filled polyurethane or vinyl chambers used to maintain a moist healing environment caused wounds to heal with less inflammation and less scarring compared to dry, air-exposed wounds^[158–160]^. Additionally, occlusive dressings, which maintain hydration, reduce both inflammation and scarring^[161]^. Occlusion reduces the production pro-inflammatory cytokines and lipids by epidermal keratinocytes, the release of pro-inflammatory alarmin molecules such as S100A8, S100A9, and S100A12, and the infiltration of inflammatory cells^[90,162–165]^. It is likely, then, that maintaining hydration mitigates scar formation in part by diminishing inflammation.

#### Mechanoregulation

Fibroblast behavior is known to be influenced by mechanical signals, and wounds that heal under tension typically heal with more scar tissue. Data from animal models have illustrated this concept, as devices which increase mechanical loading on a wound increase scar formation^[28,166]^. Mechanically-loaded wounds have also been shown to have a stronger inflammatory response^[28,29,93]^. In addition, it has been suggested that higher levels of mechanical strain in keloid tissue is associated with inflammation and may contribute to keloid progression^[167]^. Focal adhesion kinase (FAK) signaling, which controls mechanosignaling in fibroblasts, has been suggested to mediate the enhanced inflammatory reaction associated with mechanical strain^[93,167]^. Mechanosensing has also been shown in recent studies to regulate inflammatory cell migration using sophisticated *in vitro* approaches^[168]^. Here, the authors demonstrated that deformations in collagen matrices caused by contractile activity of fibroblasts provides a strong signal for macrophage migration.

Several approaches have been tested to target mechanosignaling as a way to prevent or reduce scarring. A stress-shielding device, which reduces the tension on a healing wound, has been shown to improve scarring in large animal pre-clinical models as well as human clinical trials^[166,169,170]^. Microarray studies have shown that this device reduces the transcription of genes related to inflammatory pathways^[171]^. Another strategy to reduce scar formation by affecting mechanotransduction is to inhibit FAK signaling with a small molecule inhibitor. Several small animal studies have suggested that FAK inhibitors can reduce scarring^[93,172]^, and treatment with FAK inhibitors and genetic ablation of FAK in fibroblasts have been reported to reduce inflammatory mediator production and inflammatory cell recruitment^[93]^. Collectively, the data suggest that altering mechanical signaling pathways may reduce scar formation at least in part by influencing inflammation.

## CONCLUSION

**Figure 2.**
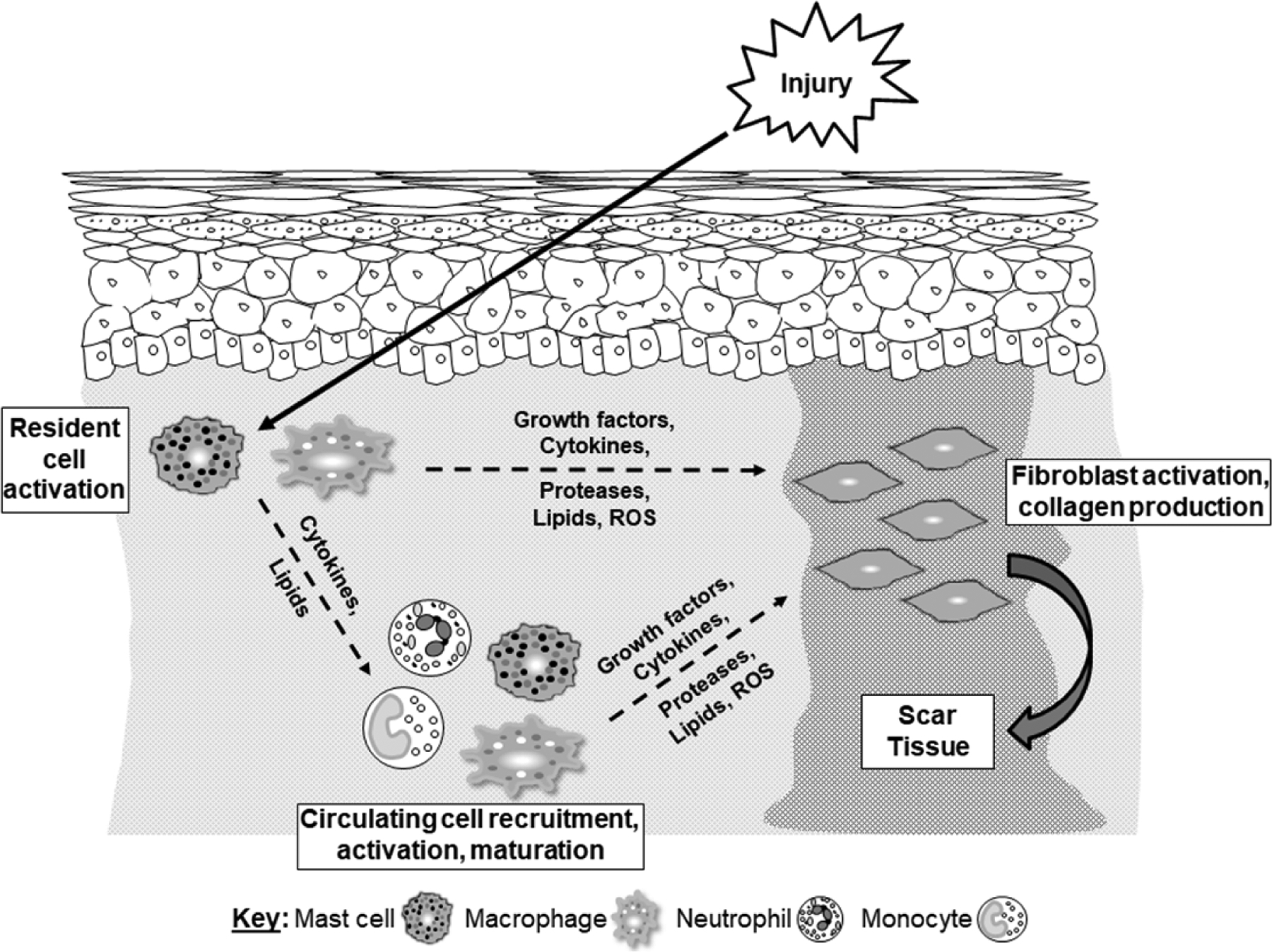
Summary of cellular interactions leading to cutaneous scar formation. When the skin is damaged, an inflammatory response is induced. Initially, resident inflammatory cells (e.g., mast cells and macrophages) in the dermis are activated. These activated cells secrete molecules that stimulate fibroblast activity and promote collagen production and scar tissue deposition. The resident cell-derived mediators, particularly cytokines (and chemokines) as well as lipids, stimulate the recruitment of circulating inflammatory cells (e.g., neutrophils, monocytes, and mast cell precursors) into the tissue. These cells become activated, and in some cases mature (monocytes become macrophages and mast cell precursors become mature mast cells), leading to even higher local levels of mediators that stimulate fibroblast activity and perpetuate scar tissue production. Several types of mediators produced by inflammatory cells have been linked to scar formation, including growth factors (TGF-β1), cytokines/chemokines [interleukin (IL)-6, IL-17, IL-33, MCP-1, SDF-1, OPN], proteases (mast cell chymase/tryptase, neutrophil elastase), lipids (PGE_2_), and reactive oxygen species or ROS (H_2_O_2_)

There is a large body of evidence suggesting that the magnitude of the inflammatory response influences the amount of scar tissue that will result from the healing process [Figure 2]. This likely results, at least in part, from the array of mediators released by inflammatory cells capable of stimulating fibroblast activity. Studies examining human tissue and samples from animal models suggest that pro-inflammatory cytokines and the number of inflammatory cells (e.g., mast cells and macrophages) are elevated in problematic scars such as HTS and keloids whereas the inflammatory response is muted in scarless wounds. Functional data support these correlative results as depleting inflammatory cells or reducing pro-inflammatory mediators generally reduces scar formation. Collectively, these data support the idea that targeting inflammation could be useful for limiting scar formation. Indeed, there are several studies, mainly using animal models, that show anti-inflammatory compounds reduce scar formation, and several other potential anti-scar treatment modalities have documented anti-inflammatory effects.

Despite the large number of studies on inflammation and scar formation, more research is needed to fully understand this relationship. Conflicting data from some studies demonstrates the complexity of the relationship between inflammation and scar formation and highlights the lack of standardized approaches for studying this relationship experimentally. In particular, variability in experimental models can make it difficult to draw broad conclusions; these models include the use of human tissue, large animal models (e.g., pigs, which have similar skin anatomy to human), and small animal models (e.g., mice, which have loose skin, but can be used for advanced functional studies). More precise characterization of inflammation that includes an expanded view of different inflammatory cell types as well as the specific subtypes of each cell and how changes in these cells over time affect scar formation are needed, especially in human specimens. In addition, further investigation is needed to determine how beneficial various anti-inflammatory approaches could be for minimizing scar formation in human skin.

## References

[1] Eming SA, Martin P, Tomic-Canic M. Wound repair and regeneration: mechanisms, signaling, and translation. Sci Transl Med 2014;6:265sr6. 25473038 10.1126/scitranslmed.3009337 PMC4973620

[2] Gurtner GC, Werner S, Barrandon Y, Longaker MT. Wound repair and regeneration. Nature 2008;453:314–21. 18480812 10.1038/nature07039

[3] Martin P. Wound healing--aiming for perfect skin regeneration. Science 1997;276:75–81. 9082989 10.1126/science.276.5309.75

[4] Aarabi S, Longaker MT, Gurtner GC. Hypertrophic scar formation following burns and trauma: new approaches to treatment. PLoS Med 2007;4:e234. 17803351 10.1371/journal.pmed.0040234 PMC1961631

[5] Corr DT, Gallant-Behm CL, Shrive NG, Hart DA. Biomechanical behavior of scar tissue and uninjured skin in a porcine model. Wound Repair Regen 2009;17:250–9. 19320894 10.1111/j.1524-475X.2009.00463.x

[6] Dunn MG, Silver FH, Swann DA. Mechanical analysis of hypertrophic scar tissue: structural basis for apparent increased rigidity. J Invest Dermatol 1985;84:9–13. 3965583 10.1111/1523-1747.ep12274528

[7] Brown BC, McKenna SP, Siddhi K, McGrouther DA, Bayat A. The hidden cost of skin scars: quality of life after skin scarring. J Plast Reconstr Aesthet Surg 2008;61:1049–58. 18617450 10.1016/j.bjps.2008.03.020

[8] Wilgus TA. Immune cells in the healing skin wound: influential players at each stage of repair. Pharmacol Res 2008;58:112–6. 18723091 10.1016/j.phrs.2008.07.009

[9] Carlsson AH, Rose LF, Fletcher JL, Wu JC, Leung KP, Antecedent thermal injury worsens split-thickness skin graft quality: a clinically relevant porcine model of full-thickness burn, excision and grafting. Burns 2017;43:223–31. 27600980 10.1016/j.burns.2016.08.006

[10] Jabeen S, Clough ECS, Thomlinson AM, Chadwick SL, Ferguson MWJ, Partial thickness wound: does mechanism of injury influence healing? Burns 2019;45:531–42. 30739729 10.1016/j.burns.2018.08.010

[11] Morris MW Jr, Allukian M 3rd, Herdrich BJ, Caskey RC, Zgheib C, Modulation of the inflammatory response by increasing fetal wound size or interleukin-10 overexpression determines wound phenotype and scar formation. Wound Repair Regen 2014;22:406–14. 24844340 10.1111/wrr.12180

[12] Dunkin CS, Pleat JM, Gillespie PH, Tyler MP, Roberts AH, Scarring occurs at a critical depth of skin injury: precise measurement in a graduated dermal scratch in human volunteers. Plast Reconstr Surg 2007;119:1722–32; discussion 1733–4. 17440346 10.1097/01.prs.0000258829.07399.f0

[13] Qian LW, Fourcaudot AB, Yamane K, You T, Chan RK, Exacerbated and prolonged inflammation impairs wound healing and increases scarring. Wound Repair Regen 2016;24:26–34. 26562746 10.1111/wrr.12381

[14] Zhang Q, Yamaza T, Kelly AP, Shi S, Wang S, Tumor-like stem cells derived from human keloid are governed by the inflammatory niche driven by IL-17/IL-6 axis. PLoS One 2009;4:e7798. 19907660 10.1371/journal.pone.0007798 PMC2771422

[15] Zhang M, Xu Y, Liu Y, Cheng Y, Zhao P, Chemokine-like factor 1 (CKLF-1) is overexpressed in keloid patients: a potential indicating factor for keloid-predisposed individuals. Medicine (Baltimore) 2016;95:e3082. 26986142 10.1097/MD.0000000000003082 PMC4839923

[16] Jumper N, Hodgkinson T, Paus R, Bayat A. Site-specific gene expression profiling as a novel strategy for unravelling keloid disease pathobiology. PLoS One 2017;12:e0172955. 28257480 10.1371/journal.pone.0172955 PMC5336271

[17] Dong X, Zhang C, Ma S, Wen H. Mast cell chymase in keloid induces profibrotic response via transforming growth factor-beta1/Smad activation in keloid fibroblasts. Int J Clin Exp Pathol 2014;7:3596–607. 25120737 PMC4128972

[18] Craig SS, DeBlois G, Schwartz LB. Mast cells in human keloid, small intestine, and lung by an immunoperoxidase technique using a murine monoclonal antibody against tryptase. Am J Pathol 1986;124:427–35. 3532813 PMC1888358

[19] Boyce DE, Ciampolini J, Ruge F, Murison MS, Harding KG. Inflammatory-cell subpopulations in keloid scars. Br J Plast Surg 2001;54:511–6. 11513514 10.1054/bjps.2001.3638

[20] Jiao H, Fan J, Cai J, Pan B, Yan L, Analysis of characteristics similar to autoimmune disease in keloid patients. Aesthetic Plast Surg 2015;39:818–25. 26296635 10.1007/s00266-015-0542-4

[21] Shaker SA, Ayuob NN, Hajrah NH. Cell talk: a phenomenon observed in the keloid scar by immunohistochemical study. Appl Immunohistochem Mol Morphol 2011;19:153–9. 20881838 10.1097/PAI.0b013e3181efa2ef

[22] Arbi S, Eksteen EC, Oberholzer HM, Taute H, Bester MJ. Premature collagen fibril formation, fibroblast-mast cell interactions and mast cell-mediated phagocytosis of collagen in keloids. Ultrastruct Pathol 2015;39:95–103. 25569098 10.3109/01913123.2014.981326

[23] Bagabir R, Byers RJ, Chaudhry IH, Müller W, Paus R, Site-specific immunophenotyping of keloid disease demonstrates immune upregulation and the presence of lymphoid aggregates. Br J Dermatol 2012;167:1053–66. 23106354 10.1111/j.1365-2133.2012.11190.x

[24] Novak ML, Koh TJ. Macrophage phenotypes during tissue repair. J Leukoc Biol 2013;93:875–81. 23505314 10.1189/jlb.1012512 PMC3656331

[25] Theoret CL, Olutoye OO, Parnell LK, Hicks J. Equine exuberant granulation tissue and human keloids: a comparative histopathologic study. Vet Surg 2013;42:783–9. 24015864 10.1111/j.1532-950X.2013.12055.x

[26] Gaber MA, Seliet IA, Ehsan NA, Megahed MA. Mast cells and angiogenesis in wound healing. Anal Quant Cytopathol Histpathol 2014;36:32–40. 24902369

[27] Hellström M, Hellström S, Engström-Laurent A, Bertheim U. The structure of the basement membrane zone differs between keloids, hypertrophic scars and normal skin: a possible background to an impaired function. J Plast Reconstr Aesthet Surg 2014;67:1564–72. 25037500 10.1016/j.bjps.2014.06.014

[28] Aarabi S, Bhatt KA, Shi Y, Paterno J, Chang EI, Mechanical load initiates hypertrophic scar formation through decreased cellular apoptosis. FASEB J 2007;21:3250–61. 17504973 10.1096/fj.07-8218com

[29] Wong VW, Paterno J, Sorkin M, Glotzbach JP, Levi K, Mechanical force prolongs acute inflammation via T-cell-dependent pathways during scar formation. FASEB J 2011;25:4498–510. 21911593 10.1096/fj.10-178087

[30] Wang J, Ding J, Jiao H, Honardoust D, Momtazi M, Human hypertrophic scar-like nude mouse model: characterization of the molecular and cellular biology of the scar process. Wound Repair Regen 2011;19:274–85. 21362096 10.1111/j.1524-475X.2011.00672.x

[31] Zhu Z, Ding J, Ma Z, Iwashina T, Tredget EE. The natural behavior of mononuclear phagocytes in HTS formation. Wound Repair Regen 2016;24:14–25. 26519112 10.1111/wrr.12378

[32] Ibrahim MM, Bond J, Bergeron A, Miller KJ, Ehanire T, A novel immune competent murine hypertrophic scar contracture model: a tool to elucidate disease mechanism and develop new therapies. Wound Repair Regen 2014;22:755–64. 25327261 10.1111/wrr.12238 PMC4304906

[33] Harunari N, Zhu KQ, Armendariz RT, Deubner H, Muangman P, Histology of the thick scar on the female, red Duroc pig: final similarities to human hypertrophic scar. Burns 2006;32:669–77. 16905264 10.1016/j.burns.2006.03.015 PMC2878281

[34] Kischer CW, Bunce H 3rd, Shetlah MR. Mast cell analyses in hypertrophic scars, hypertrophic scars treated with pressure and mature scars. J Invest Dermatol 1978;70:355–7. 649983 10.1111/1523-1747.ep12543553

[35] Beer TW, Baldwin H, West L, Gallagher PJ, Wright DH. Mast cells in pathological and surgical scars. Br J Ophthalmol 1998;82:691–4. 9797674 10.1136/bjo.82.6.691 PMC1722623

[36] Niessen FB, Schalkwijk J, Vos H, Timens W. Hypertrophic scar formation is associated with an increased number of epidermal Langerhans cells. J Pathol 2004;202:121–9. 14694529 10.1002/path.1502

[37] van den Broek LJ, van der Veer WM, de Jong EH, Gibbs S, Niessen FB. Suppressed inflammatory gene expression during human hypertrophic scar compared to normotrophic scar formation. Exp Dermatol 2015;24:623–9. 25939875 10.1111/exd.12739

[38] Butzelaar L, Schooneman DP, Soykan EA, Talhout W, Ulrich MM, Inhibited early immunologic response is associated with hypertrophic scarring. Exp Dermatol 2016;25:797–804. 27249786 10.1111/exd.13100

[39] Moore AL, Marshall CD, Barnes LA, Murphy MP, Ransom RC, Scarless wound healing: transitioning from fetal research to regenerative healing. Wiley Interdiscip Rev Dev Biol 2018;7:e309. 10.1002/wdev.309 PMC6485243 29316315

[40] Wilgus TA. Regenerative healing in fetal skin: a review of the literature. Ostomy Wound Manage 2007;53:16–31. 17586870

[41] Wilgus TA. Fetal wound healing In: Bagchi D, Das A, Roy S, editors. Wound healing, tissue repair, and regeneration in diabetes. Cambridge, MA: Academic Press; 2020 pp. 579–91.

[42] Armstrong JR, Ferguson MW. Ontogeny of the skin and the transition from scar-free to scarring phenotype during wound healing in the pouch young of a marsupial, Monodelphis domestica. Dev Biol 1995;169:242–60. 7750642 10.1006/dbio.1995.1141

[43] Kumta S, Ritz M, Hurley JV, Crowe D, Romeo R, Acute inflammation in foetal and adult sheep: the response to subcutaneous injection of turpentine and carrageenan. Br J Plast Surg 1994;47:360–8. 8087375 10.1016/0007-1226(94)90096-5

[44] Wilgus TA, Bergdall VK, Tober KL, Hill KJ, Mitra S, The impact of cyclooxygenase-2 mediated inflammation on scarless fetal wound healing. Am J Pathol 2004;165:753–61. 15331400 10.1016/S0002-9440(10)63338-X PMC1618587

[45] Liechty KW, Adzick NS, Crombleholme TM. Diminished interleukin 6 (IL-6) production during scarless human fetal wound repair. Cytokine 2000;12:671–6. 10843743 10.1006/cyto.1999.0598

[46] Liechty KW, Crombleholme TM, Cass DL, Martin B, Adzick NS. Diminished interleukin-8 (IL-8) production in the fetal wound healing response. J Surg Res 1998;77:80–4. 9698538 10.1006/jsre.1998.5345

[47] Wulff BC, Pappa NK, Wilgus TA. Interleukin-33 encourages scar formation in murine fetal skin wounds. Wound Repair Regen 2019;27:19–28. 30368969 10.1111/wrr.12687 PMC6448156

[48] Dardenne AD, Wulff BC, Wilgus TA. The alarmin HMGB-1 influences healing outcomes in fetal skin wounds. Wound Repair Regen 2013;21:282–91. 23438257 10.1111/wrr.12028 PMC3594575

[49] Gordon A, Kozin ED, Keswani SG, Vaikunth SS, Katz AB, Permissive environment in postnatal wounds induced by adenoviral-mediated overexpression of the anti-inflammatory cytokine interleukin-10 prevents scar formation. Wound Repair Regen 2008;16:70–9. 18086289 10.1111/j.1524-475X.2007.00326.x

[50] Wulff BC, Yu L, Parent AE, Wilgus TA. Novel differences in the expression of inflammation-associated genes between mid- and late-gestational dermal fibroblasts. Wound Repair Regen 2013;21:103–12. 23126606 10.1111/j.1524-475X.2012.00860.x PMC3540136

[51] Olutoye OO, Alaish SM, Carr ME, Paik M, Yager DR, Aggregatory characteristics and expression of the collagen adhesion receptor in fetal porcine platelets. J Pediatr Surg 1995;30:1649–53. 8749915 10.1016/0022-3468(95)90443-3

[52] Olutoye O, Yager D, Cohen I, Diegelmann R. Lower cytokine release by fetal porcine platelets: a possible explanation for reduced inflammation after fetal wounding. J Pediatr Surg 1996;31:91–5. 8632294 10.1016/s0022-3468(96)90326-7

[53] Wulff BC, Parent AE, Meleski MA, DiPietro LA, Schrementi ME, Mast cells contribute to scar formation during fetal wound healing. J Invest Dermatol 2012;132:458–65. 21993557 10.1038/jid.2011.324 PMC3258379

[54] Wulff BC, Wilgus TA. Examining the role of mast cells in fetal wound healing using cultured cells *in vitro* In: Gourdie RG, Myers TA, editors. Wound regeneration and repair. Totowa: Humana Press; 2013 pp. 495–506. 10.1007/978-1-62703-505-7_29 24029955

[55] Cowin A, Brosnan M, Holmes T, Ferguson M. Endogenous inflammatory response to dermal wound healing in the fetal and adult mouse. Dev Dyn 1998;212:385–93. 9671942 10.1002/(SICI)1097-0177(199807)212:3<385::AID-AJA6>3.0.CO;2-D

[56] Hopkinson-Woolley J, Hughes D, Gordon S, Martin P. Macrophage recruitment during limb development and wound healing in the embryonic and foetal mouse. J Cell Sci 1994;107 (Pt 5):1159–67. 7929625 10.1242/jcs.107.5.1159

[57] Burrington JD. Wound healing in the fetal lamb. J Pediatr Surg 1971;6:523–8. 5166565 10.1016/0022-3468(71)90373-3

[58] Naik-Mathuria B, Gay AN, Yu L, Hsu JE, Smith CW, Fetal wound healing using a genetically modified murine model: the contribution of P-selectin. J Pediatr Surg 2008;43:675–82. 18405715 10.1016/j.jpedsurg.2007.12.007 PMC2424112

[59] Olutoye OO, Zhu X, Cass DL, Smith CW. Neutrophil recruitment by fetal porcine endothelial cells: implications in scarless fetal wound healing. Pediatr Res 2005;58:1290–4. 16306210 10.1203/01.pdr.0000184326.01884.bc

[60] Rowlatt U. Intrauterine wound healing in a 20 week human fetus. Virchows Arch A Pathol Anat Histol 1979;381:353–61. 155931 10.1007/BF00432477

[61] Walraven M, Talhout W, Beelen RH, van Egmond M, Ulrich MM. Healthy human second-trimester fetal skin is deficient in leukocytes and associated homing chemokines. Wound Repair Regen 2016;24:533–41. 26873861 10.1111/wrr.12421

[62] Wong JW, Gallant-Behm C, Wiebe C, Mak K, Hart DA, Wound healing in oral mucosa results in reduced scar formation as compared with skin: evidence from the red Duroc pig model and humans. Wound Repair Regen 2009;17:717–29. 19769724 10.1111/j.1524-475X.2009.00531.x

[63] Szpaderska AM, Zuckerman JD, DiPietro LA. Differential injury responses in oral mucosal and cutaneous wounds. J Dent Res 2003;82:621–6. 12885847 10.1177/154405910308200810

[64] Mak K, Manji A, Gallant-Behm C, Wiebe C, Hart DA, Scarless healing of oral mucosa is characterized by faster resolution of inflammation and control of myofibroblast action compared to skin wounds in the red Duroc pig model. J Dermatol Sci 2009;56:168–80. 19854029 10.1016/j.jdermsci.2009.09.005

[65] Glim JE, Beelen RH, Niessen FB, Everts V, Ulrich MM. The number of immune cells is lower in healthy oral mucosa compared to skin and does not increase after scarring. Arch Oral Biol 2015;60:272–81. 25463905 10.1016/j.archoralbio.2014.10.008

[66] Chen L, Arbieva ZH, Guo S, Marucha PT, Mustoe TA, Positional differences in the wound transcriptome of skin and oral mucosa. BMC Genomics 2010;11:471. 20704739 10.1186/1471-2164-11-471 PMC3091667

[67] Iglesias-Bartolome R, Uchiyama A, Molinolo AA, Abusleme L, Brooks SR, Transcriptional signature primes human oral mucosa for rapid wound healing. Sci Transl Med 2018;10:eaap8798. 30045979 10.1126/scitranslmed.aap8798 PMC6598699

[68] Seifert AW, Kiama SG, Seifert MG, Goheen JR, Palmer TM, Skin shedding and tissue regeneration in African spiny mice (Acomys). Nature 2012;489:561–5. 23018966 10.1038/nature11499 PMC3480082

[69] Brant JO, Lopez MC, Baker HV, Barbazuk WB, Maden M. A comparative analysis of gene expression profiles during skin regeneration in Mus and Acomys. PLoS One 2015;10:e0142931. 26606282 10.1371/journal.pone.0142931 PMC4659537

[70] Brant JO, Yoon JH, Polvadore T, Barbazuk WB, Maden M. Cellular events during scar-free skin regeneration in the spiny mouse, Acomys. Wound Repair Regen 2016;24:75–88. 26606280 10.1111/wrr.12385

[71] Dabrowski R, Drobnik J. The effect of disodium cromoglycate on the skin wound healing and collagen content in the wounds of rats. Acta Physiol Pol 1990;41:195–8. 2136192

[72] Chen L, Schrementi ME, Ranzer MJ, Wilgus TA, DiPietro LA. Blockade of mast cell activation reduces cutaneous scar formation. PLoS One 2014;9:e85226. 24465509 10.1371/journal.pone.0085226 PMC3898956

[73] Gallant-Behm CL, Hildebrand KA, Hart DA. The mast cell stabilizer ketotifen prevents development of excessive skin wound contraction and fibrosis in red Duroc pigs. Wound Repair Regen 2008;16:226–33. 18318808 10.1111/j.1524-475X.2008.00363.x

[74] Younan GJ, Heit YI, Dastouri P, Kekhia H, Xing W, Mast cells are required in the proliferation and remodeling phases of microdeformational wound therapy. Plast Reconstr Surg 2011;128:649e–58. 10.1097/PRS.0b013e318230c55d 22094766

[75] Shiota N, Nishikori Y, Kakizoe E, Shimoura K, Niibayashi T, Pathophysiological role of skin mast cells in wound healing after scald injury: study with mast cell-deficient W/W(V) mice. Int Arch Allergy Immunol 2010;151:80–8. 19672099 10.1159/000232573

[76] Antsiferova M, Martin C, Huber M, Feyerabend TB, Förster A, Mast cells are dispensable for normal and activin-promoted wound healing and skin carcinogenesis. J Immunol 2013;191:6147–55. 24227781 10.4049/jimmunol.1301350

[77] Nauta AC, Grova M, Montoro DT, Zimmermann A, Tsai M, Evidence that mast cells are not required for healing of splinted cutaneous excisional wounds in mice. PLoS One 2013;8:e59167. 23544053 10.1371/journal.pone.0059167 PMC3609818

[78] Willenborg S, Eckes B, Brinckmann J, Krieg T, Waisman A, Genetic ablation of mast cells redefines the role of mast cells in skin wound healing and bleomycin-induced fibrosis. J Invest Dermatol 2014;134:2005–15. 24406680 10.1038/jid.2014.12

[79] Wulff BC, Wilgus TA. Mast cell activity in the healing wound: more than meets the eye? Exp Dermatol 2013;22:507–10. 23802591 10.1111/exd.12169 PMC3723719

[80] Martin P, D’souza D, Martin J, Grose R, Cooper L, Wound healing in the PU.1 null mouse-tissue repair is not dependent on inflammatory cells. Curr Biol 2003;13:1122–8. 12842011 10.1016/s0960-9822(03)00396-8

[81] Chrysanthopoulou A, Mitroulis I, Apostolidou E, Arelaki S, Mikroulis D, Neutrophil extracellular traps promote differentiation and function of fibroblasts. J Pathol 2014;233:294–307. 24740698 10.1002/path.4359

[82] Mirza R, DiPietro LA, Koh TJ. Selective and specific macrophage ablation is detrimental to wound healing in mice. Am J Pathol 2009;175:2454–62. 19850888 10.2353/ajpath.2009.090248 PMC2789630

[83] Lucas T, Waisman A, Ranjan R, Roes J, Krieg T, Differential roles of macrophages in diverse phases of skin repair. J Immunol 2010;184:3964–77. 20176743 10.4049/jimmunol.0903356

[84] Rodero MP, Legrand JM, Bou-Gharios G, Khosrotehrani K. Wound-associated macrophages control collagen 1α2 transcription during the early stages of skin wound healing. Exp Dermatol 2013;22:143–5. 23278967 10.1111/exd.12068

[85] Zhu Z, Ding J, Ma Z, Iwashina T, Tredget EE. Systemic depletion of macrophages in the subacute phase of wound healing reduces hypertrophic scar formation. Wound Repair Regen 2016;24:644–56. 27169512 10.1111/wrr.12442

[86] Shook BA, Wasko RR, Rivera-Gonzalez GC, Salazar-Gatzimas E, López-Giráldez F, Myofibroblast proliferation and heterogeneity are supported by macrophages during skin repair. Science 2018;362:eaar2971. 30467144 10.1126/science.aar2971 PMC6684198

[87] Sinha M, Sen CK, Singh K, Das A, Ghatak S, Direct conversion of injury-site myeloid cells to fibroblast-like cells of granulation tissue. Nat Commun 2018;9:936. 29507336 10.1038/s41467-018-03208-w PMC5838200

[88] Jeong W, Yang CE, Roh TS, Kim JH, Lee JH, Scar prevention and enhanced wound healing induced by polydeoxyribonucleotide in a rat incisional wound-healing model. Int J Mol Sci 2017;18:1698. 10.3390/ijms18081698 PMC5578088 28771195

[89] Wilgus TA, Vodovotz Y, Vittadini E, Clubbs EA, Oberyszyn TM. Reduction of scar formation in full-thickness wounds with topical celecoxib treatment. Wound Repair Regen 2003;11:25–34. 12581424 10.1046/j.1524-475x.2003.11106.x

[90] Xu W, Hong SJ, Zeitchek M, Cooper G, Jia S, Hydration status regulates sodium flux and inflammatory pathways through epithelial sodium channel (ENaC) in the skin. J Invest Dermatol 2015;135:796–806. 25371970 10.1038/jid.2014.477

[91] Zhang J, Qiao Q, Liu M, He T, Shi J, IL-17 promotes scar formation by inducing macrophage infiltration. Am J Pathol 2018;188:1693–702. 29753790 10.1016/j.ajpath.2018.04.005

[92] Ferreira AM, Takagawa S, Fresco R, Zhu X, Varga J, Diminished induction of skin fibrosis in mice with MCP-1 deficiency. J Invest Dermatol 2006;126:1900–8. 16691201 10.1038/sj.jid.5700302

[93] Wong VW, Rustad KC, Akaishi S, Sorkin M, Glotzbach JP, Focal adhesion kinase links mechanical force to skin fibrosis via inflammatory signaling. Nat Med 2011;18:148–52. 22157678 10.1038/nm.2574 PMC4457506

[94] Cooper L, Johnson C, Burslem F, Martin P. Wound healing and inflammation genes revealed by array analysis of ‘macrophageless’ PU.1 null mice. Genome Biol 2005;6:R5. 15642097 10.1186/gb-2004-6-1-r5 PMC549066

[95] Mori R, Shaw TJ, Martin P. Molecular mechanisms linking wound inflammation and fibrosis: knockdown of osteopontin leads to rapid repair and reduced scarring. J Exp Med 2008;205:43–51. 18180311 10.1084/jem.20071412 PMC2234383

[96] Liechty KW, Kim HB, Adzick NS, Crombleholme TM. Fetal wound repair results in scar formation in interleukin-10-deficient mice in a syngeneic murine model of scarless fetal wound repair. J Pediatr Surg 2000;35:866–72; discussion 872–3. 10873028 10.1053/jpsu.2000.6868

[97] Kieran I, Knock A, Bush J, So K, Metcalfe A, Interleukin-10 reduces scar formation in both animal and human cutaneous wounds: results of two preclinical and phase II randomized control studies. Wound Repair Regen 2013;21:428–36. 23627460 10.1111/wrr.12043

[98] Wise LM, Stuart GS, Jones NC, Fleming SB, Mercer AA. Orf virus IL-10 and VEGF-E act synergistically to enhance healing of cutaneous wounds in mice. J Clin Med 2020;9:1085. 10.3390/jcm9041085 PMC7231296 32290480

[99] Wise LM, Stuart GS, Real NC, Fleming SB, Mercer AA. Orf virus IL-10 accelerates wound healing while limiting inflammation and scarring. Wound Repair Regen 2014;22:356–67. 24844335 10.1111/wrr.12169

[100] Balaji S, King A, Marsh E, LeSaint M, Bhattacharya SS, The role of interleukin-10 and hyaluronan in murine fetal fibroblast function in vitro: implications for recapitulating fetal regenerative wound healing. PLoS One 2015;10:e0124302. 25951109 10.1371/journal.pone.0124302 PMC4423847

[101] Balaji S, Wang X, King A, Le LD, Bhattacharya SS, Interleukin-10-mediated regenerative postnatal tissue repair is dependent on regulation of hyaluronan metabolism via fibroblast-specific STAT3 signaling. FASEB J 2017;31:868–81. 27903619 10.1096/fj.201600856R PMC5295728

[102] King A, Balaji S, Le LD, Marsh E, Crombleholme TM, Interleukin-10 regulates fetal extracellular matrix hyaluronan production. J Pediatr Surg 2013;48:1211–7. 23845609 10.1016/j.jpedsurg.2013.03.014 PMC3959980

[103] King A, Balaji S, Marsh E, Le LD, Shaaban AF, Interleukin-10 regulates the fetal hyaluronan-rich extracellular matrix via a STAT3-dependent mechanism. J Surg Res 2013;184:671–7. 23684616 10.1016/j.jss.2013.04.009 PMC3759570

[104] Caskey RC, Allukian M, Lind RC, Herdrich BJ, Xu J, Lentiviral-mediated over-expression of hyaluronan synthase-1 (HAS-1) decreases the cellular inflammatory response and results in regenerative wound repair. Cell Tissue Res 2013;351:117–25. 23149717 10.1007/s00441-012-1504-7

[105] Estes JM, Scott Adzick N, Harrison MR, Longaker MT, Stern R. Hyaluronate metabolism undergoes and ontogenic transition during fetal development: Implications for Scar-free wound healing. J Pediatr Surg 1993;28:1227–31. 8263679 10.1016/s0022-3468(05)80303-3

[106] Longaker MT, Scott Adzick N, Hall JL, Stair SE, Crombleholme TM, Studies in fetal wound healing, VII. Fetal wound healing may be modulated by hyaluronic acid stimulating activity in amniotic fluid. J Pediatr Surg 1990;25:430–3. 2329459 10.1016/0022-3468(90)90387-o

[107] Longaker MT, Chiu ES, Adzick NS, Stern M, Harrison MR, Studies in fetal wound healing. V. A prolonged presence of hyaluronic acid characterizes fetal wound fluid. Ann Surg 1991;213:292–6. 2009010 10.1097/00000658-199104000-00003 PMC1358347

[108] Longaker MT, Chiu ES, Harrison MR, Crombleholme TM, Langer JC, Studies in fetal wound healing. IV. Hyaluronic acid-stimulating activity distinguishes fetal wound fluid from adult wound fluid. Ann Surg 1989;210:667–72. 2818035 10.1097/00000658-198911000-00016 PMC1357805

[109] Longaker MT, Harrison MR, Crombleholme TM, Langer JC, Decker M, Studies in fetal wound healing: I. A factor in fetal serum that stimulates deposition of hyaluronic acid. J Pediatr Surg 1989;24:789–92. 2769547 10.1016/s0022-3468(89)80538-x

[110] West DC, Shaw DM, Lorenz P, Adzick NS, Longaker MT. Fibrotic healing of adult and late gestation fetal wounds correlates with increased hyaluronidase activity and removal of hyaluronan. Int J Biochem Cell Biol 1997;29:201–10. 9076955 10.1016/s1357-2725(96)00133-1

[111] Yates CC, Krishna P, Whaley D, Bodnar R, Turner T, Lack of CXC chemokine receptor 3 signaling leads to hypertrophic and hypercellular scarring. Am J Pathol 2010;176:1743–55. 20203286 10.2353/ajpath.2010.090564 PMC2843466

[112] Cash JL, Bass MD, Campbell J, Barnes M, Kubes P, Resolution mediator chemerin15 reprograms the wound microenvironment to promote repair and reduce scarring. Curr Biol 2014;24:1406–14. 24881877 10.1016/j.cub.2014.05.006 PMC4064685

[113] Sidgwick GP, McGeorge D, Bayat A. A comprehensive evidence-based review on the role of topicals and dressings in the management of skin scarring. Arch Dermatol Res 2015;307:461–77. 26044054 10.1007/s00403-015-1572-0 PMC4506744

[114] Amini-Nik S, Yousuf Y, Jeschke MG. Scar management in burn injuries using drug delivery and molecular signaling: Current treatments and future directions. Adv Drug Deliv Rev 2018;123:135–54. 28757325 10.1016/j.addr.2017.07.017 PMC5742037

[115] Ogawa R, Akita S, Akaishi S, Aramaki-Hattori N, Dohi T, Diagnosis and treatment of keloids and hypertrophic scars-japan scar workshop consensus document 2018. Burns Trauma 2019;7:39. 31890718 10.1186/s41038-019-0175-y PMC6933735

[116] Roques C, Téot L. The use of corticosteroids to treat keloids: a review. Int J Low Extrem Wounds 2008;7:137–45. 18611924 10.1177/1534734608320786

[117] Kim DY, Han YS, Kim SR, Chun BK, Park JH. Effects of a topical angiotensin-converting enzyme inhibitor and a selective COX-2 inhibitor on the prevention of hypertrophic scarring in the skin of a rabbit ear. Wounds 2012;24:356–64. 25876220

[118] Yuan Z, Zhao J, Chen Y, Yang Z, Cui W, Regulating inflammation using acid-responsive electrospun fibrous scaffolds for skin scarless healing. Mediators Inflamm 2014;2014:858045. 24795507 10.1155/2014/858045 PMC3984856

[119] Rahmani-Neishaboor E, Jallili R, Hartwell R, Leung V, Carr N, Topical application of a film-forming emulgel dressing that controls the release of stratifin and acetylsalicylic acid and improves/prevents hypertrophic scarring. Wound Repair Regen 2013;21:55–65. 23126516 10.1111/j.1524-475X.2012.00857.x

[120] Medina A, Ghaffari A, Kilani RT, Ghahary A. The role of stratifin in fibroblast-keratinocyte interaction. Mol Cell Biochem 2007;305:255–64. 17646930 10.1007/s11010-007-9538-y

[121] Rahmani-Neishaboor E, Yau FM, Jalili R, Kilani RT, Ghahary A. Improvement of hypertrophic scarring by using topical anti-fibrogenic/anti-inflammatory factors in a rabbit ear model. Wound Repair Regen 2010;18:401–8. 20546553 10.1111/j.1524-475X.2010.00598.x

[122] Wilgus TA. Alerting the body to tissue injury: the role of alarmins and DAMPs in cutaneous wound healing. Curr Pathobiol Rep 2018;6:55–60. 29862143 10.1007/s40139-018-0162-1 PMC5978745

[123] Wang J, Hori K, Ding J, Huang Y, Kwan P, Toll-like receptors expressed by dermal fibroblasts contribute to hypertrophic scarring. J Cell Physiol 2011;226:1265–73. 20945369 10.1002/jcp.22454

[124] Li XP, Liu P, Li YF, Zhang GL, Zeng DS, LPS induces activation of the TLR4 pathway in fibroblasts and promotes skin scar formation through collagen I and TGF-beta in skin lesions. Int J Clin Exp Pathol 2019;12:2121–9. 31934034 PMC6949634

[125] Bhattacharyya S, Tamaki Z, Wang W, Hinchcliff M, Hoover P, FibronectinEDA promotes chronic cutaneous fibrosis through Toll-like receptor signaling. Sci Transl Med 2014;6:232ra50. 10.1126/scitranslmed.3008264 PMC4414050 24739758

[126] Bhattacharyya S, Wang W, Qin W, Cheng K, Coulup S, TLR4-dependent fibroblast activation drives persistent organ fibrosis in skin and lung. JCI Insight 2018;3:98850. 29997297 10.1172/jci.insight.98850 PMC6124522

[127] Bhattacharyya S, Wang W, Tamaki Z, Shi B, Yeldandi A, Pharmacological inhibition of Toll-like receptor-4 signaling by TAK242 prevents and induces regression of experimental organ fibrosis. Front Immunol 2018;9:2434. 30405628 10.3389/fimmu.2018.02434 PMC6207051

[128] Blohm-Mangone K, Burkett NB, Tahsin S, Myrdal PB, Aodah A, Pharmacological TLR4 antagonism using topical resatorvid blocks solar UV-induced skin tumorigenesis in SKH-1 mice. Cancer Prev Res (Phila) 2018;11:265–78. 29437671 10.1158/1940-6207.CAPR-17-0349 PMC5932085

[129] Janda J, Burkett NB, Blohm-Mangone K, Huang V, Curiel-Lewandrowski C, Resatorvid-based pharmacological antagonism of cutaneous TLR4 blocks UV-induced NF-κB and AP-1 signaling in keratinocytes and mouse skin. Photochem Photobiol 2016;92:816–25. 27859308 10.1111/php.12659 PMC5161657

[130] Rice TW, Wheeler AP, Bernard GR, Vincent JL, Angus DC, A randomized, double-blind, placebo-controlled trial of TAK-242 for the treatment of severe sepsis. Crit Care Med 2010;38:1685–94. 20562702 10.1097/CCM.0b013e3181e7c5c9

[131] Ding J, Hori K, Zhang R, Marcoux Y, Honardoust D, Stromal cell-derived factor 1 (SDF-1) and its receptor CXCR4 in the formation of postburn hypertrophic scar (HTS). Wound Repair Regen 2011;19:568–78. 22092795 10.1111/j.1524-475X.2011.00724.x

[132] Liu H, Ding J, Ma Z, Zhu Z, Shankowsky HA, A novel subpopulation of peripheral blood mononuclear cells presents in major burn patients. Burns 2015;41:998–1007. 25683215 10.1016/j.burns.2014.12.005

[133] Shin JU, Kim SH, Kim H, Noh JY, Jin S, TSLP is a potential initiator of collagen synthesis and an activator of CXCR4/SDF-1 axis in keloid pathogenesis. J Invest Dermatol 2016;136:507–15. 26824743 10.1016/j.jid.2015.11.008

[134] Hall CL, Wells AR, Leung KP. Pirfenidone reduces profibrotic responses in human dermal myofibroblasts, in vitro. Lab Invest 2018;98:640–55. 29497173 10.1038/s41374-017-0014-3

[135] Saito M, Yamazaki M, Maeda T, Matsumura H, Setoguchi Y, Pirfenidone suppresses keloid fibroblast-embedded collagen gel contraction. Arch Dermatol Res 2012;304:217–22. 22033529 10.1007/s00403-011-1184-2

[136] Wells AR, Leung KP. Pirfenidone attenuates the profibrotic contractile phenotype of differentiated human dermal myofibroblasts. Biochem Biophys Res Commun 2020;521:646–51. 31679692 10.1016/j.bbrc.2019.10.177

[137] Satish L, Evdokiou A, Geletu E, Hahn JM, Supp DM. Pirfenidone inhibits epithelial-mesenchymal transition in keloid keratinocytes. Burns Trauma 2020;8:tkz007. 32405508 10.1093/burnst/tkz007 PMC7175767

[138] Dorati R, Medina JL, DeLuca PP, Leung KP. Development of a topical 48-H release formulation as an anti-scarring treatment for deep partial-thickness burns. AAPS PharmSciTech 2018;19:2264–75. 29790019 10.1208/s12249-018-1030-3

[139] Medina JL, Sebastian EA, Fourcaudot AB, Dorati R, Leung KP. Pirfenidone ointment modulates the burn wound bed in c57bl/6 mice by suppressing inflammatory responses. Inflammation 2019;42:45–53. 30120654 10.1007/s10753-018-0871-y

[140] Armendariz-Borunda J, Lyra-Gonzalez I, Medina-Preciado D, Gonzalez-García I, Martinez-Fong D, A controlled clinical trial with pirfenidone in the treatment of pathological skin scarring caused by burns in pediatric patients. Ann Plast Surg 2012;68:22–8. 21659848 10.1097/SAP.0b013e31821b6d08

[141] Afaq F, Adhami VM, Ahmad N, Mukhtar H. Inhibition of ultraviolet B-mediated activation of nuclear factor kappaB in normal human epidermal keratinocytes by green tea Constituent (−)-epigallocatechin-3-gallate. Oncogene 2003;22:1035–44. 12592390 10.1038/sj.onc.1206206

[142] Elmets CA, Singh D, Tubesing K, Matsui M, Katiyar S, Cutaneous photoprotection from ultraviolet injury by green tea polyphenols. J Am Acad Dermatol 2001;44:425–32. 11209110 10.1067/mjd.2001.112919

[143] Gensler HL, Timmermann BN, Valcic S, Wächter GA, Dorr R, Prevention of photocarcinogenesis by topical administration of pure epigallocatechin gallate isolated from green tea. Nutr Cancer 1996;26:325–35. 8910914 10.1080/01635589609514488

[144] Katiyar SK, Mukhtar H. Green tea polyphenol (−)-epigallocatechin-3-gallate treatment to mouse skin prevents UVB-induced infiltration of leukocytes, depletion of antigen-presenting cells, and oxidative stress. J Leukoc Biol 2001;69:719–26. 11358979

[145] Zhang Q, Kelly AP, Wang L, French SW, Tang X, Green tea extract and (−)-epigallocatechin-3-gallate inhibit mast cell-stimulated type I collagen expression in keloid fibroblasts via blocking PI-3K/AkT signaling pathways. J Invest Dermatol 2006;126:2607–13. 16841034 10.1038/sj.jid.5700472

[146] Park G, Yoon BS, Moon JH, Kim B, Jun EK, Green tea polyphenol epigallocatechin-3-gallate suppresses collagen production and proliferation in keloid fibroblasts via inhibition of the STAT3-signaling pathway. J Invest Dermatol 2008;128:2429–41. 18463684 10.1038/jid.2008.103

[147] Syed F, Bagabir RA, Paus R, Bayat A. Ex vivo evaluation of antifibrotic compounds in skin scarring: EGCG and silencing of PAI-1 independently inhibit growth and induce keloid shrinkage. Lab Invest 2013;93:946–60. 23835737 10.1038/labinvest.2013.82

[148] Ud-Din S, Foden P, Mazhari M, Al-Habba S, Baguneid M, A double-blind, randomized trial shows the role of zonal priming and direct topical application of epigallocatechin-3-gallate in the modulation of cutaneous scarring in human skin. J Invest Dermatol 2019;139:1680–90.e16. 30822414 10.1016/j.jid.2019.01.030

[149] Soo C, Hu F, Zhang X, Wang Y, Beanes SR, Differential expression of fibromodulin, a transforming growth factor-β modulator, in fetal skin development and scarless repair. The American Journal of Pathology 2000;157:423–33. 10934147 10.1016/s0002-9440(10)64555-5 PMC1850122

[150] Honardoust D, Varkey M, Hori K, Ding J, Shankowsky HA, Small leucine-rich proteoglycans, decorin and fibromodulin, are reduced in postburn hypertrophic scar. Wound Repair Regen 2011;19:368–78. 21518082 10.1111/j.1524-475X.2011.00677.x

[151] Honardoust D, Varkey M, Marcoux Y, Shankowsky HA, Tredget EE. Reduced decorin, fibromodulin, and transforming growth factor-β3 in deep dermis leads to hypertrophic scarring. J Burn Care Res 2012;33:218–27. 22079916 10.1097/BCR.0b013e3182335980

[152] Zheng Z, Lee KS, Zhang X, Nguyen C, Hsu C, Fibromodulin-deficiency alters temporospatial expression patterns of transforming growth factor-β ligands and receptors during adult mouse skin wound healing. PLoS One 2014;9:e90817. 24603701 10.1371/journal.pone.0090817 PMC3948369

[153] Zheng Z, Zhang X, Dang C, Beanes S, Chang GX, Fibromodulin is essential for fetal-type scarless cutaneous wound healing. Am J Pathol 2016;186:2824–32. 27665369 10.1016/j.ajpath.2016.07.023 PMC5222972

[154] Zheng Z, Nguyen C, Zhang X, Khorasani H, Wang JZ, Delayed wound closure in fibromodulin-deficient mice is associated with increased TGF-β3 signaling. J Invest Dermatol 2011;131:769–78. 21191417 10.1038/jid.2010.381 PMC4073663

[155] Jiang W, Ting K, Lee S, Zara JN, Song R, Fibromodulin reduces scar size and increases scar tensile strength in normal and excessive-mechanical-loading porcine cutaneous wounds. J Cell Mol Med 2018;22:2510–3. 29392829 10.1111/jcmm.13516 PMC5867110

[156] Stoff A, Rivera AA, Mathis JM, Moore ST, Banerjee NS, Effect of adenoviral mediated overexpression of fibromodulin on human dermal fibroblasts and scar formation in full-thickness incisional wounds. J Mol Med (Berl) 2007;85:481–96. 17219096 10.1007/s00109-006-0148-z

[157] Pang X, Dong N, Zheng Z. Small leucine-rich proteoglycans in skin wound healing. Front Pharmacol 2019;10:1649. 32063855 10.3389/fphar.2019.01649 PMC6997777

[158] Breuing K, Eriksson E, Liu P, Miller DR. Healing of partial thickness porcine skin wounds in a liquid environment. J Surg Res 1992;52:50–8. 1548868 10.1016/0022-4804(92)90278-8

[159] Junker JP, Kamel RA, Caterson EJ, Eriksson E. Clinical impact upon wound healing and inflammation in moist, wet, and dry environments. Adv Wound Care (New Rochelle) 2013;2:348–56. 24587972 10.1089/wound.2012.0412 PMC3842869

[160] Reish RG, Zuhaili B, Bergmann J, Aflaki P, Koyama T, Modulation of scarring in a liquid environment in the Yorkshire pig. Wound Repair Regen 2009;17:806–16. 19903302 10.1111/j.1524-475X.2009.00546.x

[161] Mustoe TA, Gurjala A. The role of the epidermis and the mechanism of action of occlusive dressings in scarring. Wound Repair Regen 2011;19 Suppl 1:s16–21. 21793961 10.1111/j.1524-475X.2011.00709.x PMC3725331

[162] Gallant-Behm CL, Mustoe TA. Occlusion regulates epidermal cytokine production and inhibits scar formation. Wound Repair Regen 2010;18:235–44. 20419876 10.1111/j.1524-475x.2010.00575.x PMC2860621

[163] Zhao J, Zhong A, Friedrich EE, Jia S, Xie P, S100A12 induced in the epidermis by reduced hydration activates dermal fibroblasts and causes dermal fibrosis. J Invest Dermatol 2017;137:650–9. 27840235 10.1016/j.jid.2016.10.040

[164] Zhong A, Xu W, Zhao J, Xie P, Jia S, S100A8 and S100A9 are induced by decreased hydration in the epidermis and promote fibroblast activation and fibrosis in the dermis. Am J Pathol 2016;186:109–22. 26597884 10.1016/j.ajpath.2015.09.005

[165] O’Shaughnessy KD, De La Garza M, Roy NK, Mustoe TA. Homeostasis of the epidermal barrier layer: a theory of how occlusion reduces hypertrophic scarring. Wound Repair Regen 2009;17:700–8. 19769722 10.1111/j.1524-475X.2009.00534.x

[166] Gurtner GC, Dauskardt RH, Wong VW, Bhatt KA, Wu K, Improving cutaneous scar formation by controlling the mechanical environment: large animal and phase I studies. Ann Surg 2011;254:217–25. 21606834 10.1097/SLA.0b013e318220b159

[167] Dohi T, Padmanabhan J, Akaishi S, Than PA, Terashima M, The interplay of mechanical stress, strain, and stiffness at the keloid periphery correlates with increased caveolin-1/ROCK signaling and scar progression. Plast Reconstr Surg 2019;144:58e–67. 10.1097/PRS.0000000000005717 31246819

[168] Pakshir P, Alizadehgiashi M, Wong B, Coelho NM, Chen X, Dynamic fibroblast contractions attract remote macrophages in fibrillar collagen matrix. Nat Commun 2019;10:1850. 31015429 10.1038/s41467-019-09709-6 PMC6478854

[169] Lim AF, Weintraub J, Kaplan EN, Januszyk M, Cowley C, The embrace device significantly decreases scarring following scar revision surgery in a randomized controlled trial. Plast Reconstr Surg 2014;133:398–405. 24105084 10.1097/01.prs.0000436526.64046.d0 PMC4874339

[170] Longaker MT, Rohrich RJ, Greenberg L, Furnas H, Wald R, A randomized controlled trial of the embrace advanced scar therapy device to reduce incisional scar formation. Plast Reconstr Surg 2014;134:536–46. 24804638 10.1097/PRS.0000000000000417 PMC4425293

[171] Januszyk M, Wong VW, Bhatt KA, Vial IN, Paterno J, Mechanical offloading of incisional wounds is associated with transcriptional downregulation of inflammatory pathways in a large animal model. Organogenesis 2014;10:186–93. 24739276 10.4161/org.28818 PMC4154952

[172] Ma K, Kwon SH, Padmanabhan J, Duscher D, Trotsyuk AA, Controlled delivery of a focal adhesion kinase inhibitor results in accelerated wound closure with decreased scar formation. J Invest Dermatol 2018;138:2452–60. 29775632 10.1016/j.jid.2018.04.034

